# Non-pharmacological interventions for older adults with mild cognitive impairment: an umbrella review

**DOI:** 10.1186/s12877-026-07933-6

**Published:** 2026-08-13

**Authors:** Diana C. Tiga-Loza, Edwing A. Urrea-Vega, Raquel Rivera-Carvajal, William A. Alvarez-Anaya, Jhosman A. Buitrago-Buitrago, Ana M. Hernández-Martínez, Paul A. Camacho-López

**Affiliations:** 1 Universidad de Santander, Institito MASIRA, Facultad de Ciencias Médicas y de La Salud, Bucaramanga, COL Colombia; 2 Universidad de Santander, Doctorado en Enfermedades Infecciosas, Facultad de ciencias médicas y de la salud, Bucaramanga, COL Colombia; 3 Programa de Enfermería, Universidad Manuela Beltrán, Bucaramanga, COL Colombia; 4 Dirección de Investigaciones, Universidad Manuela Beltrán, Bucaramanga, COL Colombia; 5 Universidad de Santander, Grupo de investigación en fisioterapia integral, Facultad de ciencias médicas y de la salud, Bucaramanga, Colombia; 6 Departamento de Investigación, Desarrollo E Innovación Tecnológica, Fundación Oftalmológica de Santander (FOSCAL), Floridablanca, COL Colombia

**Keywords:** Older adult, Cognitive Decline, Intervention, Multicomponent

## Abstract

**Background:**

Mild cognitive impairment (MCI) is an intermediate stage between normal aging and dementia in which preventive interventions may help delay cognitive decline. Multicomponent non-pharmacological interventions (NPTs) combining physical, cognitive, and psychosocial components have shown promising results; however, the available evidence remains heterogeneous.

**Objective:**

Synthesize evidence from systematic reviews and meta-analyses (SRMAs) evaluating multicomponent, non-pharmacological interventions designed to improve cognitive function among community-dwelling older adults with MCI.

**Methods:**

An umbrella review was conducted following PRISMA, PRIOR, and Joanna Briggs Institute recommendations. Searches were performed in Web of Science, Embase, PubMed, BIREME, Cochrane Library, and Google Scholar for systematic reviews published between 2012 and 2025. Methodological quality was assessed using AMSTAR 2 and certainty of evidence using GRADE. A narrative synthesis was performed due to methodological heterogeneity.

**Results:**

Sixteen reviews were included, comprising five systematic reviews (SRs) and eleven systematic reviews with meta-analyses (SRMAs). Across these reviews, 322 randomized controlled trials were reported. Of these, 143 met eligibility criteria, yielding 84 unique primary trials after overlap removal (CCA = 4.68%), comprising 5,818 participants. Cognitive-physical combined interventions predominated. Median participant age was 74.8 years (IQR 71.7–77.6), session duration 60 min (IQR 45–92.5), frequency 2 sessions/week (IQR 2–3), and program length 16 weeks (IQR 10–24). Memory (57.1%), executive function (54.1%), global cognition (47.1%), and attention (42.9%) were the domains most frequently reported as showing statistically significant benefits. These proportions reflect the frequency of positive findings across trials rather than pooled effect estimates and should be interpreted cautiously given the heterogeneity and methodological variability of the included evidence.

**Conclusions:**

Multicomponent non-pharmacological interventions may provide cognitive benefits, particularly for memory and executive function, though certainty varied and findings from lower-quality reviews should be interpreted cautiously.

**Supplementary Information:**

The online version contains supplementary material available at https://doi.org/10.1186/s12877-026-07933-6.

## Background

Aging is a progressive and dynamic biological process that affects physical health, cognitive functioning, and emotional well-being. The global increase in life expectancy, together with demographic transitions, has made population aging a worldwide phenomenon with profound implications for social and economic systems [1, 2]. This shift has been one of the major global challenges since the twentieth century and is characterized by the inversion of population pyramids [3].

According to the World Health Organization (WHO), the number of older adults is expected to double worldwide by 2050. Latin America is projected to experience particularly rapid growth, with the proportion of older adults rising from 11% in 2018 to approximately 25% within 35 years, about half the time it took Europe to reach a similar level [4, 5]. This demographic trend has contributed to a higher prevalence of cognitive impairment and neurodegenerative disorders, including Alzheimer’s disease (AD) and other dementias, which in turn increases disability and dependence among older adults [6, 7]. It is estimated that around 150 million people worldwide will be living with dementia by 2050 [8].

According to the National Institute on Aging and Alzheimer’s Association (NIA-AA) framework, Mild Cognitive Impairment (MCI) is a symptomatic transitional state between normal aging and dementia. It is characterized by objective evidence of decline in one or more cognitive domains beyond what would be expected for an individual’s age and education level. Although these deficits may reduce efficiency in complex daily tasks, functional independence is preserved; therefore, the individual does not meet clinical criteria for dementia [9]. Despite its conceptual clarity, applying the diagnosis of MCI in routine clinical practice remains challenging [10].

The global prevalence of MCI has been estimated at 19.7% (95% CI: 18.3–21.1) based on a recent systematic review and meta-analysis. Prevalence varies by setting and is higher in hospitals (34.0%; 95% CI: 22.2–45.7) than in nursing homes (22.6%; 95% CI: 15.5–29.8) and community-based samples (17.9%; 95% CI: 16.6–19.2) [11]. Although some individuals remain stable or even improve over time, 34–50% of MCI cases progress to dementia. In people with metabolic syndrome, the risk of progression is almost tripled (OR = 2.95; 95% CI: 1.23–7.05 [12], and sarcopenia has also been identified as an independent risk factor for MCI (OR = 1.46; 95% CI: 1.31–1.62) [13].

In its 2020 report, The Lancet Commission on Dementia Prevention, Intervention, and Care identified 12 modifiable risk factors and estimated a population attributable fraction of 56% in Latin America, suggesting substantial potential for prevention, particularly in low- and middle-income countries [14]. These risk factors span the life course: early life (education), midlife (hypertension, obesity, hearing loss, traumatic brain injury, and alcohol consumption), and later life (smoking, depression, physical inactivity, social isolation, diabetes, and air pollution) [14].

The commission also recommends multicomponent interventions as the preferred approach to mitigate neuropsychiatric symptoms in people with dementia [14]. In this review, the term “multicomponent” is used to encompass interventions described in the literature as multidomain or multimodal. In routine care, management of MCI typically emphasizes etiologic assessment and longitudinal monitoring, including evaluation of potentially modifiable risk factors and neuropsychiatric symptoms, review of medications that may impair cognition, and follow-up of cognitive and functional status over time [15–17].

Lifestyle modifications and non-pharmacological strategies, such as aerobic exercise, have shown promising effects in delaying progression from MCI to dementia [18, 19]. A wide range of interventions has been studied to preserve or improve cognition in older adults, including game-based cognitive training [20], dance therapy [21], combined or sequential cognitive–motor programs (exergaming) [22, 23], yoga [24], psychosocial and lifestyle interventions [25], arts-based approaches such as music, drama, or visual arts [26] and emerging mobile application–based tools [27].

Current evidence suggests that multicomponent (multimodal or multidomain) interventions are generally more effective than single-domain approaches for enhancing cognitive performance in older adults [28]. However, the optimal combination, frequency, intensity, and duration of these interventions remain uncertain, and the literature shows considerable heterogeneity in study designs and outcomes. Given this variability and the growing number of systematic reviews on the topic, an umbrella review is warranted. This approach enables a comprehensive synthesis of high-level evidence, helping clinicians and policymakers interpret findings across broad thematic areas [29].

Therefore, the present study aims to synthesize evidence from systematic reviews and meta-analyses (SRMAs) evaluating multicomponent, non-pharmacological interventions designed to improve cognitive function among community-dwelling older adults with MCI.

## Methods

An umbrella review of systematic reviews of randomized controlled trials (RCTs) was performed and registered in the International Prospective Registry of Systematic Reviews (PROSPERO) CRD42022326619, protocol version 1.2 published on 07 May 2025.

This umbrella review was conducted in accordance with the Preferred Reporting Items for Overviews of Reviews (PRIOR) statement [30], as well as the Joanna Briggs Institute (JBI) methodology for overviews of reviews [31]. A completed PRIOR Checklist is provided as Supplemental Material 1 to ensure transparency and adherence to reporting standards. The PRISMA standards definition of systematic review was used.

### Literature search and inclusion criteria

A comprehensive and standardized search strategy was applied to Web of Science, Embase, PubMed, BIREME, and the Cochrane Library to identify eligible SRMAs published between January 2012 and June 2025. The final literature search update was conducted in June 2025. Additionally, the first 100 results from Google Scholar were screened, and a snowballing approach was applied to identify relevant references cited in included reviews.

The review focused on older adults diagnosed with mild cognitive impairment (MCI). Although the search period was initiated in 2012 to align with the publication and broader adoption of the 2011 National Institute on Aging–Alzheimer’s Association (NIA-AA) criteria, eligibility was not restricted exclusively to studies applying the NIA-AA framework. Systematic reviews were considered eligible when the primary studies included participants diagnosed with MCI according to recognized clinical or research diagnostic criteria, including NIA-AA, Petersen criteria, DSM classifications, or comparable standardized definitions reported by the original reviews [9].

The full search strategy, including Medical Subject Headings (MeSH) and free-text terms (e.g., “Mild Cognitive Impairment,” “non-pharmacological interventions,” “older adults”), is provided in Supplemental Material 2.

The research question was formulated according to the PICO framework (see Supplemental Material 3), Population: community-dwelling older adults with MCI; Intervention: multicomponent non-pharmacological interventions (e.g., cognitive training, physical exercise, nutrition counseling, psychosocial programs); Comparator: standard care or single-domain interventions; Outcome: cognitive function and related neuropsychological domains. The guiding question was: “What is the effect of multicomponent non-pharmacological interventions on cognitive function among older adults with MCI?”.

#### Inclusion criteria

SRs or SRMAs based on RCTs that evaluated multicomponent non-pharmacological interventions addressing lifestyle modification, physical or cognitive training, nutritional counseling, psychosocial activities, or complementary mind–body therapies. Systematic reviews including mixed populations (e.g., participants with mild cognitive impairment and dementia) were eligible only when data specific to the MCI subgroup could be clearly identified, extracted separately, or interpreted independently from the overall synthesis. During data extraction, reviewers assessed whether participant characteristics, subgroup analyses, or outcome reporting allowed isolation of evidence corresponding specifically to individuals with MCI. Reviews in which MCI-specific findings could not be reasonably distinguished from dementia-related outcomes were excluded from the synthesis.

The review was restricted to systematic reviews involving community-dwelling older adults with MCI, explicitly excluding institutionalized or hospitalized cohorts. This selection was intended to prioritize interventions with ecological validity, focusing on those whose frequency and delivery mechanisms (e.g., home-based or community-center sessions) are feasible within a real-world, non-clinical framework. By targeting non-institutionalized individuals, this review ensures that the synthesized evidence on multicomponent interventions is directly generalized to public health initiatives and primary care settings.

#### Exclusion criteria

Scoping and narrative reviews; non-experimental studies; animal and in vitro research; interventions involving pharmacological agents such as cholinesterase inhibitors (donepezil, rivastigmine, galantamine) or NMDA antagonists (memantine); hormonal therapies; or nutritional supplements (e.g., vitamins, minerals, amino acids, fatty acids, or protein powders). Also excluded were editorials, review protocols without results, reviews of single-domain interventions, studies without cognitive outcomes, or those involving only participants with subjective cognitive decline or dementia.

### Data extraction (selection and coding)

Reference management and screening were conducted using Rayyan QCRI (Qatar Computing Research Institute, QCRI; www.rayyan.ai). Rayyan is a web-based platform that facilitates semi-automated screening and selection of records in systematic reviews. It employs text-mining and machine-learning algorithms to support the identification of eligible studies based on inclusion and exclusion criteria. By allowing real-time collaboration among reviewers and highlighting conflicts or duplicates, Rayyan enhances transparency, reproducibility, and efficiency in the screening process. This substantial screening efficiency reduces reviewer workload while maintaining methodological rigor, making it a valuable tool for evidence synthesis in umbrella and systematic reviews [32].

Duplicate records were automatically removed. Two reviewer pairs (RRC, EU, DT, MM and JB) independently and blindly screened titles and abstracts. Disagreements were resolved through discussion and consensus after unblinding. Full texts were reviewed against inclusion criteria, and reasons for exclusion are listed in Supplemental Material 4.

Data extraction was performed using a structured template in Microsoft Excel that captured the following variables: publication details, population characteristics, number of included RCTs (k) and participants (n), intervention domains and descriptions, setting, comparators, outcome measures, statistical methods, and main findings. Quantitative data extracted included relative risk (RR), odds ratio (OR), hazard ratio (HR), confidence intervals (95% CI), and effect sizes (e.g., Cohen’s d), as well as heterogeneity metrics (Cochran’s Q, I^2^, Egger’s test) and *p*-values for pooled estimates. Additionally, qualitative data included methodological limitations, quality appraisals, and authors’ conclusions.

### Assessment of the methodological quality of the meta-analyses reviewed

Two reviewers (EU and JB) independently assessed methodological quality using the AMSTAR 2 (A Measurement Tool to Assess Systematic Reviews) checklist, classifying reviews as high, moderate, low, or critically low quality (See Supplemental Material 5).

Certainty of evidence was evaluated by two independent reviewers (D. T. and E. U.) using the Grading of Recommendations, Assessment, Development, and Evaluation (GRADE) approach. GRADE assessments were conducted at the level of the included systematic reviews, based on the evidence profiles and methodological judgments reported by their authors; no independent regrading of primary trials was performed. These assessments considered the domains of risk of bias, inconsistency, indirectness, and imprecision, and were categorized as high, moderate, low, or very low certainty [33, 34]. For systematic reviews providing exclusively narrative syntheses without pooled quantitative estimates, several GRADE domains requiring quantitative precision measures (e.g., inconsistency, imprecision, and publication bias) could not be formally assessed and were therefore reported as “not applicable” (N/A). Alternative certainty frameworks such as GRADE-CERQual were not applied because the included reviews synthesized narrative summaries of randomized controlled trials rather than qualitative evidence. This limitation was considered during interpretation of the findings. Detailed results are provided in Supplemental Material 5.

### Data synthesis and analysis

Given the methodological and clinical heterogeneity among the included SRMAs, a narrative (qualitative) synthesis was conducted. Descriptive analyses were performed using a Microsoft Excel matrix to summarize characteristics of the SRMAs and their primary RCTs. Qualitative variables are presented as frequencies and percentages, whereas continuous variables (e.g., mean age, session duration) are summarized using medians and interquartile ranges (IQRs), prioritizing information disaggregated by intervention and control group and resorting to the overall mean only when data by group were unavailable.

The primary unit of analysis was the systematic review with or without meta-analysis; primary RCTs were examined solely for contextualization and were not reanalyzed quantitatively. Study overlap among the included reviews was assessed using the corrected covered area (CCA) to estimate the potential risk of double-counting of primary studies. The level of overlap was interpreted according to the criteria proposed by Pieper and Kirvalidze [35, 36], where CCA values of 0–5 indicate slight overlap, 6–10 moderate overlap, 11–15 high overlap, and > 15 very high overlap. The observed CCA value was 4.68%, indicating slight overlap among the included reviews.

The CCA was calculated using the following formula:

$$CCA=\frac{\left(N-r\right)}{\left(rc-r\right)}$$

where:

*N*: = Total number of studies included in the systematic reviews (including duplicates).

*r*: = Number of unique primary studies (excluding duplicates).

*c*: = Number of systematic reviews included in the umbrella review.

Despite the low overlap identified, an additional quantitative meta-analysis at the umbrella review level was not performed because of substantial heterogeneity in intervention characteristics, cognitive outcome measures, follow-up duration, and synthesis approaches across the included reviews. Therefore, a structured narrative synthesis was considered the most appropriate approach for evidence integration.

Findings were interpreted according to a pre-specified evidence hierarchy. Greater interpretive weight was assigned to outcomes from reviews rated as high or moderate methodological quality (AMSTAR 2) with moderate or high certainty of evidence (GRADE). Findings from low or critically low-quality reviews, or outcomes with low or very low certainty, were treated as hypothesis-generating only. Consistency across reviews, magnitude of effect, heterogeneity, and overlap were also considered when appraising the overall strength of inference.

An inductive thematic coding process was applied to standardize cognitive and behavioral outcomes into conceptual domains. For example, the “memory domain” included working memory, delayed recall, and episodic memory outcomes, whereas the “physical activity domain” grouped aerobic, resistance, and mind–body interventions such as Tai Chi or yoga.

## Results

**Fig. 1:**
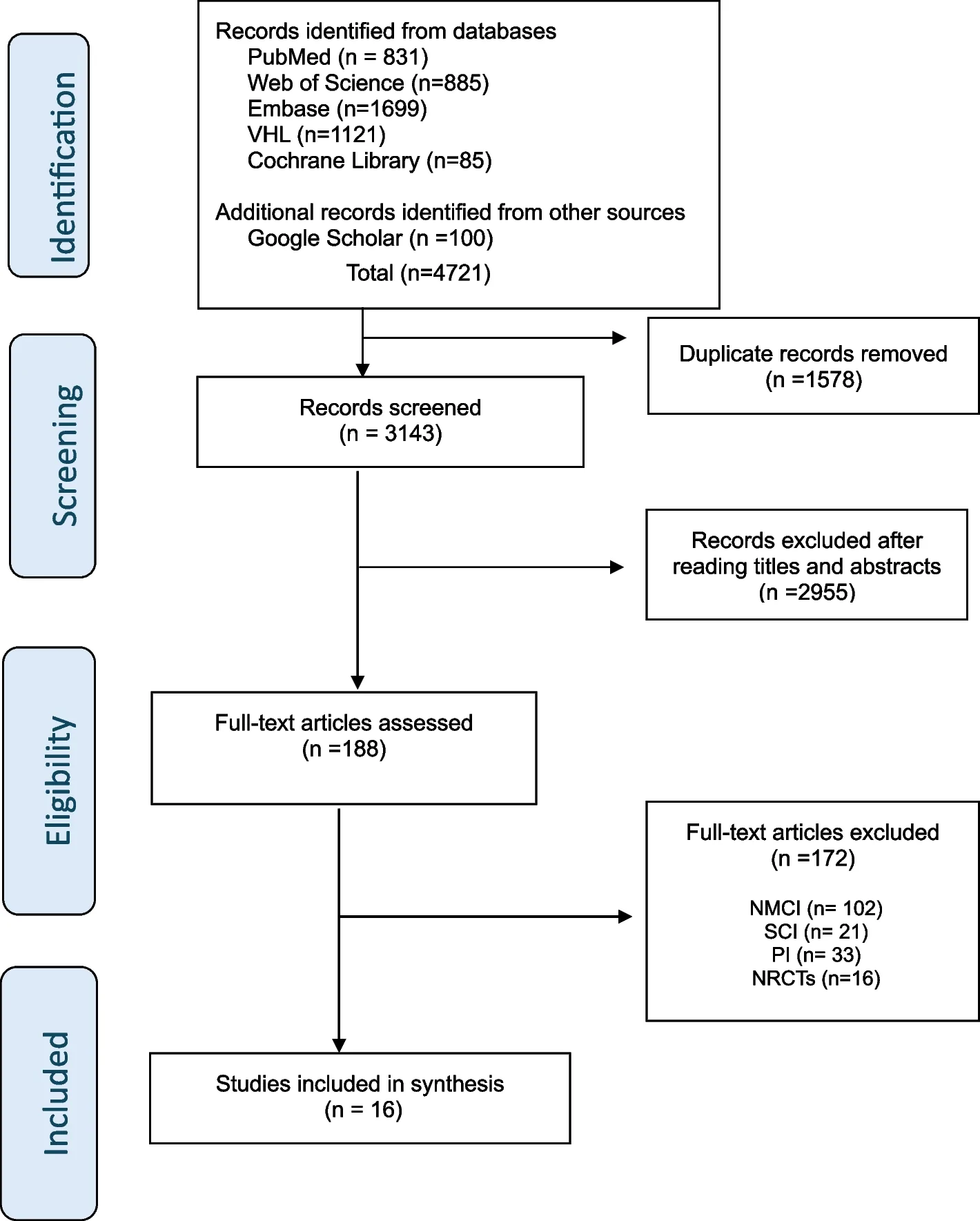
PRISMA Flow Diagram. VHL = Virtual Health Library, NMCI = Non-Multicomponent Intervention, SCI = Subjective Cognitive Impairment, PI = Pharmacological Intervention, NRCTs = Non-Randomized Clinical Trials

As illustrated in Fig. 1, the comprehensive search yielded a total of 4,721 records: 4,621 from electronic databases and 100 from Google Scholar. After removing 1,578 duplicates, 3,143 records were excluded based on title and abstract screening. A total of 188 full-text articles were subsequently assessed for eligibility according to the predefined inclusion criteria and the type of intervention evaluated. Of these, 172 reviews were excluded due to the inclusion of pharmacological interventions, studies based on subjective diagnoses of cognitive impairment, non-randomized designs, or single-component interventions. Consequently, 16 systematic reviews and meta-analyses met the inclusion criteria and were incorporated into qualitative synthesis.

Of the 16 reviews included, five were systematic reviews (SRs) and eleven were systematic reviews with meta-analyses (SRMAs). The first authors were affiliated with institutions in China (*n* = 5), Finland (*n* = 2), Australia (*n* = 2), Switzerland (*n* = 1), Belgium (*n* = 1), Germany (*n* = 1), the United States (*n* = 2), Malaysia (*n* = 1), and Korea (*n* = 1).

The number of original randomized controlled trials (RCTs) included in these reviews ranged from 8 to 47, totaling 322 RCTs. The mean age of participants across studies was above 70 years, which is consistent with the older adult population targeted. Detailed study characteristics are summarized in Table 1.

**Table 1:** Characteristics of the included SRs and MAs

Author	Year	Country	Total RCTs	Included		Mean age (Min; Max)	Participants	Design
				**RCT**	**Baseline N**			
Öhman et al. [37]	2014	Finland	22	4	259	76.8(72.8; 82)	MCI/Dementia	SRs
Kallio et al. [38]	2017	Finland	31	5	253	74.2(73; 77)	MCI/AD	SRs
Carrion et al. [39]	2018	Switzerland	47	7	1681	77.15(69.5; 85)	MCI/AD	SRs
Gheysen et al. [40]	2018	Belgium	41	6	725	72.09(67.7; 78.5)	MCI	SRMAs
Gates et al. [41]	2019	Australia	8	3	322	74.94(70; 79)	MCI or memory deficit or MMSE score > 24 and normal independence	SRMAs
Özbe et al. [42]	2019	Germany	9	9	521	70.3 (69.4; 84.5)	MCI/AD	SRs
Guo et al. [43]	2020	China	40	6	635	73.32(67.7; 79.1)	MCI-not have any neurological conditions (depression)	SRMAs
Wang et al. [44]	2020	China	25	4	816	73,30(68;76,3)	MCI	SRMAs
Yang et al. [45]	2020	US	10	9	1354	75.27(67.7; 87.2)	MCI	SRs
Malmberg et al. [46]	2021	Australia	41	13	941	74.97 (67; 87.2)	MCI/Dementia	SRMAs
Jung et al. [47]	2021	Korea	11	5	554	73.98(69.5; 80.9)	MCI/SMC	SRMAs
Meng et al. [48]	2021	China	16	16	1337	74.34(68.22; 86.9)	MCI/mNCD	SRMAs
Ali et al. [49]	2022	China	21	21	2221	76.25(67.5; 87.2)	MCI/AD	SRMAs
Wu et al. [50]	2023	China	21	4	246	74.25(60;92)	Healthy/MCI/Dementia	SRMAs
Qing Yi et al. [51]	2024	Malaysa	12	12	1075	71.15 (60.0; 87.0)	MCI (amnestic MCI and non-amnestic MCI)	SRMAs
Ying et al. [52]	2024	US	18	18	1555	73.54 (67.82;87.2)	MCI	SRMAs

*SRs* Systematic reviews, *SRMAs* Systematic reviews with Meta-analyses, *RCT* Randomized controlled trials, *MCI* Mild cognitive impairment, *AD* Alzheimer's Disease, *SMC* Subjective Memory Complaints, *mNCD* Mild neurocognitive disorders, *MMSE* Mini-Mental State Examination, *US* United States

Only MCI-specific findings or separately interpretable subgroup data were considered in the umbrella review synthesis

As shown in Table 2, the most frequently implemented interventions combined physical and cognitive activity (15/16, 93.8%). The median duration of the interventions ranged from 12 to 32 weeks, with individual sessions lasting between 40 and 120 min. The frequency of sessions varied between two and four times per week, resulting in a total of 24 to 100 sessions across the different intervention programs.

**Table 2:** Details of multimodal/multicomponent interventions for MCI

Author	Intervention	Duration of Intervention Median (min–max)	Contrast	Outcome	Results	Conclusions
Öhman et al. [37]	Aerobic exercise, strength training, balance, and dual tasking Tai Chi and cognitive behavioral therapy Walk and talk	Treatment duration: 32 (16–40 weeks) Session duration: 45 (30–90 min) Frequency: 3 (2–5 per week) Total sessions: 100 (48–120 sessions)	Education about health promotion Usual treatment No intervention Conversation only	Global cognition Memory Communication	Global cognition improved in studies that combine aerobic, balance, and strength exercises and dual tasking or combined Tai Chi with cognitive behavioral therapy No differences on walking and talking Memory: Physical exercise plus dual tasking improved memory according to the Wechsler Memory Scale-Logical Memory II Communication: Walking and talking improve communication performance	The use of a multicomponent program, including dual tasking, could improve global cognition and memory. The results in communication were divergent
Kallio et al. [38]	Cognitive intervention Physical intervention Combined intervention	Treatment duration: 16 (12–48 weeks) Session duration: 40 (30–90 min) Frequency: 2 (1–3 per week) Total sessions: 48 (20–103 sessions)	Psychosocial support Routine treatment	Global cognition Clinical Dementia Rating	Four of the five studies had a positive effect on global cognition and one on memory, but others reported a positive effect on affective status Multicomponent cognitive interventions were of high quality, and the frequency of intervention sessions was higher compared to single-component interventions Only one study reported no effect on cognition, and it had a small sample (14 participants)	Combined intervention is better structured and could be an option for MCI
Carrion et al. [39]	Cognitive intervention Physical interventions Combined intervention Other	Treatment duration: 18 (10–98 weeks) Session duration: 90 (60–210 min) Frequency: 4 (1–6 per week) Total sessions: 60 (13–288 sessions)	Pharmacotherapy Cognitive training Standard nursing Home care standard support Usual control No treatment	Global cognition Language/memory Executive function	Three of seven studies found significant differences in cognition after the multicomponent intervention, including improvements in global function, memory, language, and executive functioning	Multicomponent intervention can improve cognition
Gheysen et al. [40]	Cognitive intervention Physical intervention Combined intervention	Treatment duration: 16 (8–48 weeks) Session duration: 60 (30–120 min) Frequency: 3 (1–3 per week) Total sessions: 36 (20–144 sessions)	Education classes Recreational activity Physical activity	Global cognition Memory Executive functioning Other	Nine studies with 749 participants get a Hedges’ *g* = 0.389 [95% CI: 0.095–0.684] *p* =.009	In MCI participants, it showed significant positive effects
Gates et al. [41]	Cognitive intervention Physical intervention	Treatment duration: 20 (12–24 weeks) Session duration: 60 (30–75 min) Frequency: 2.5 (2–3 per week) Total sessions: 42 (32–72 sessions)	Cognitive/exercise sham/music therapy	Global cognition Memory Executive functioning Speed of processing Verbal fluency	Two studies reported SMD, one with a positive effect (SMD: -.94[−1.53, −0.35]) and the other SMD: 0.16[−0.23, 0.55] One study reported that the intervention group showed no progressive decline, unlike the control group	Computerized cognitive training combined with other interventions can help maintain cognition
Özbe et al. [42]	Cognitive interventions Physical Interventions: Combined intervention	Treatment duration: 20(6–58 weeks) Session duration: 120 (90–240 min) Frequency: 2 (1–6 per week) Total sessions: 30 (6–288 sessions)	Supervised motor placebo training, education, waitlist, paper–pencil exercises for isolated cognitive functions, and standard care	Global Cognition Memory Other	Six studies applied two, two studies applied three, and one study applied four components. Four studies, which combined at least physical and cognitive components, had a positive effect on non-cognitive symptoms of dementia. Two of these interventions additionally had a positive effect on cognitive abilities	Multicomponent Interventions suggest a good effect on MCI
Guo et al. [43]	Cognitive intervention Physical intervention Combined intervention Social activities	Treatment duration: 15 (6–48 weeks) Session duration: 50 (30–110 min) Frequency: 2 (1–3 per week) Total sessions: 45 (12–144 sessions)	Physical activity Education Passive control	Global cognition Memory Executive functioning Other	Six studies got an SSM:0.19 IC95%: 0.01 to 0.37 *p* < 0.05, I2:6.74	The combined interventions had a significant effect on MCI
Wang et al. [44]	Cognitive intervention Physical intervention Combined intervention	Treatment duration: 24(24–32 weeks) Session duration: 75 (60–90 min) Frequency: 2.5 (2–3 per week) Total sessions: 40 (20–96 sessions)	Education classes Social activity	Global cognition	Mean difference 1.66 (0.62,2.70) Heterogeneity *p* = 0.730	Non-pharmacological therapies may be effective in improving the cognition of patients with MCI
Yang et al. [45]	Cognitive interventions Physical interventions	Treatment duration: 24(6–40 weeks) Session duration: 60 (30–120 min) Frequency: 2 (1–6 per week) Total sessions: 24 (6–96 sessions)	Active control: Health promotion classes, only cognitive or physical training, Omega3 supplement Sham cognitive and exercise interventions	Global cognition Memory Executive functioning Verbal fluency Attention Finger dexterity Others Visual/spatial ability	Six (of eight) studies reported a significant improvement in global cognition with combined cognitive and physical interventions	There is evidence to support the positive effects of multicomponent interventions on patients with risk of developing dementia
Malmberg et al. [46]	Cognitive intervention Physical intervention Combined intervention	Treatment duration: 12 (6–40 weeks) Session duration: 60 (30–120 min) Frequency: 3 (1–9 per week) Total sessions: 36 (12–168 sessions)	Only exercise (cycling, balance, aerobics, and strength) Only computerized cognitive training Passive control Sham: stretching, documentaries, health education, Relaxation and flexibility	Global cognition Memory Attention Executive functioning Visual/spatial ability Finger dexterity Reaction velocity Other	Seven articles reported a positive effect on global cognition with p-values < 0.05, and two reported no significant changes. Other studies report effects on executive functions, memory, and working memory Effect on cognition: Hedges’ *g* = 0.26 (95% IC: 0.06 to 0.46). *p* = 0.02 Effect in interventions of 12 weeks or less: Hedges’ *g* = 0.24 (95% IC: 0.13 to 0.36). *p* < 0.001 Effect in supervised interventions = Hedges’ *g* = 0.23 (95% IC: 0.15 to 0.31). *p* < 0.001 Simultaneous and sequential training were significantly more efficacious for cognitive function than physical exercise (*g* = 0.24 and 0.15, respectively)	Simultaneously and sequentially supervised combined interventions are efficacious for promoting cognitive and physical health in older adults with MCI
Jung et al. [47]	Combination using Information and Communication Technology Web multidomain platform focused on three lifestyles Virtual Reality-based physical and cognitive training Interactive physical–cognitive game Virtual reality-based cognitive training Educational program	Treatment duration: 14(8–24 weeks) Session duration: 60 (20–100 min) Frequency: 3 (2–4 per week) Total sessions: 36 (24–96 sessions)	Exer-tour Only the wrist-worn accelerometer, information about multidomain activities Combined physical and cognitive training Educational material Educational program	Global cognition Memory Executive function Visual-spatial ability	The pooled SMD of the multidomain intervention was 0.53 (CI: 0.09–0.97, *p* = 0.02), and the single cognitive intervention was 0.35 (CI: 0.02–0.67, *p* = 0.04)	Multidomain intervention had a larger effect than single-domain intervention on cognitive function
Meng et al. [48]	Cognitive interventions Physical interventions Combined interventions	Treatment duration: 12 (4–40 weeks) Session duration: 52.5 (30–100 min) Frequency: 2 (1–7 per week) Total sessions: 24 (8–70 sessions)	Single cognitive intervention or single physical exercise intervention Education, watching videos, no training, usual lifestyle, usual care, and nurse-led risk factor modification	Global cognition Memory Executive function/ attention	Global cognition: Combined vs. without physical or cognitive intervention [SMD = 0.27, 95% CI (0.09, 0.44), *p* = 0.003] Memory: combined vs. single physical exercise [SMD = 0.25, 95% CI (0.07–0.44), *p* = 0.006] combined vs. without physical or cognitive intervention [SMD = 0.29, 95% CI (0.12–0.47), *p* = 0.001] Executive function/attention: combined vs. single cognitive intervention [SMD = 0.28, 95% CI (0.09–0.47), *p* = 0.004], combined vs. the single physical exercise [SMD = 0.32, 95% CI (0.16–0.49), *p* = 0.0002], and combined vs. without physical or cognitive intervention [SMD = 0.23, 95% CI (0.05–0.41), *p* = 0.01]	Combined interventions improve global cognition, memory, and executive function/attention in older adults with MCI The combined interventions demonstrated superiority over single physical exercise for memory and executive functions and single cognitive for executive function/attention
Ali et al. [49]	Cognitive interventions Physical Interventions Dual tasking interventions	Treatment duration: 24(6–52 weeks) Session duration: 60 (30–120 min) Frequency: 2 (1–8 per week) Total sessions: 40 (6–416 sessions)	Motor placebo training, Nonmusical cognitive task and walk Usual lifestyle Health education Placebo activities	Global cognitive function Memory Executive function and attention	Dual-task training showed a small-to-medium positive effect in global cognitive function; SMD = 0.24, (*p* = 0.002), memory; SMD = 0.28, (*p* = 0.000), executive function; SMD = 0.35, (*p* = 0.000) and low effect in attention; SMD = − 0.19, (*p* = 0.1)	Dual-task training (cognition and physical training)’’ had a small-to-medium positive effect on cognitive functions
Wu et al. [50]	Combined interventions (physical and cognitive)	Treatment duration: 12 (8–24 weeks) Session duration: 60 (45–90 min) Frequency: 2 (2–3 per week) Total sessions: not reported	Single cognitive intervention or single physical exercise intervention or control group	Working memory	Simultaneous/sequential multidomain intervention (Physical and cognitive) showed a medium positive effect SMD = 0.58, 95% CI [0.29–0.87], *p* < 0.01)	The combination of exercise and cognitive training is more effective than either intervention alone in working memory in MCI
Qing Yi et al. [51]	Combined interventions (Exercise and cognitive)	Treatment duration: 20 (6–48 weeks) Session duration: 73 (18–120 min) Frequency: 2 (1–3 per week) Total sessions: 35 (12—96 sessions)	Active control (health education, leisure activities, or toning exercises) or an inactive control (no-contact, waiting list, or business as usual)	Global or specific cognition domains	Combined intervention significantly enhanced global cognition, with low heterogeneity (SMD = 0.26, 95% CI [0.14–0.39], *p* < 0.0001, I2 = 0%, greatly enhanced memory, with low heterogeneity (SMD = 0.30, 95% CI [0.22–0.39], *p* < 0.00001, I^2^ = 3%)	The effects of the combined intervention on cognitive and physical function demonstrated that global cognition and majority of cognition domains were considerably enhanced when compared to the control group
Ying et al. [52]	Multimodal behavioral or cognitive interventions	Treatment duration: 22 (4—50 weeks) Session duration: 90 (30—120 min) Frequency: 2 (1–3 per week) Total sessions: 17 (16—18 sessions)	Nontreatment control groups and alternative multimodal or single modality treatment	Global cognition and mood	Multilevel meta-analyses demonstrated moderate effect (Hedge’s g = 0.44, 95% CI = [0.21–0.67]) in cognitive outcomes and large effect in mood (g = 0.65, 95% CI = [0.37–0.93]). Subdomain analyses found low-moderate effects in global cognition, verbal and non-verbal memory, executive function, visuospatial abilities, and semantic fluency (0.20 < g < 0.50)	These findings showed comparable to larger effects of multimodal cognitive and behavioral interventions on cognition than pharmacological treatment. Future studies should focus on the longitudinal effects of multimodal interventions in delaying dementia

*ADAS-Cog* Alzheimer’s Disease Assessment Scale, *AVLT* Auditory verbal learning test, *ASL* Arterial Spin Labeling, *CAMCI* Computerized Assessment of Mild Cognitive Impairment, *CDT* Clock Drawing Test, *CDR* Clinical Dementia Rating, *DRT* Disjunctive reaction time, *HVLT* Hopkins Verbal Learning Test, *IADL*-*CV* Lawton’s Instrumental Activities of Daily Living, *MRI* Magnetic Resonance Imaging, *MMSE* Mini Mental State Examination, *MoCa* Montreal Cognitive Assessment, *NTB* Neuropsychological Test Battery, *NHPT* Nine-hole peg test, *RBMT* Rivermead Behavioral Memory Test, Second Edition, *RAVLT* Rey Auditory Verbal Learning Test, *RBANS* Repeatable Battery for the Assessment of Neuropsychological Status, *SMD* Standardized mean difference, *STD* Stroop Word Color Test, *TMT* Trail-Making Test, *TEA* Test of Everyday Attention, *WMS* Wechsler Memory Scale

Values reported for intervention frequency and total session count correspond to the predominant or most consistently reported intervention protocol within each review when multiple intervention arms were available

### Quality of the articles

Table 3 summarizes the methodological quality (AMSTAR-2) and the certainty of the evidence (GRADE) for the sixteen included reviews. According to the AMSTAR-2 assessment, four reviews were rated as high quality, one as moderate, two as low, and nine as critically low quality. The domains with the lowest adherence were Item 10 (reporting of funding sources for the included primary studies), which was addressed in only one review, and Items 2 and 7 (protocol registration and provision of a list of excluded studies with reasons), which were reported in six reviews (37.5%). The remaining AMSTAR-2 domains showed satisfactory compliance, with more than 75% of the reviews meeting the corresponding criteria.

**Table 3:** Methodological quality and certainty of evidence of the included reviews according to AMSTAR-2 and GRADE

Author	Participants	AMSTAR-2	AMSTARD	Inconsistency	Indirectness	Imprecision	Publication bias	Effect Size	Gradient	Residual confounding	Grade
		**Quality**	**Risk of Bias**								**Certainty of the evidence**
Öhman et al. [37]	MCI/Dementia	Critically Low	Serious	N/A	N/A	N/A	N/A	N/A	N/A	N/A	N/A
Kallio et al. [38]	MCI/AD	Critically Low	Serious	N/A	N/A	N/A	N/A	N/A	N/A	N/A	N/A
Carrion et al. [39]	MCI/AD	Critically Low	Serious	N/A	N/A	N/A	N/A	N/A	N/A	N/A	N/A
Gheysen et al. [40]	MCI	High	Not serious	Not serious	Not serious	Serious	Yes	Medium	Yes	Yes	High ⊕ ⊕ ⊕ ⊕
Gates et al. [41]	MCI or memory deficit or MMSE score > 24 and normal independence	High	Not serious	Very serious	Serious	Serious	Not reported	Ignored	Yes	Yes	Moderate ⊕ ⊕ ⊕
Özbe et al. [42]	MCI/AD	Low	Serious	Very serious	Very serious	Very serious	Not reported	Ignored	Not	Not	Very low ⊕
Guo et al. [43]	MCI-not have any neurological conditions (depression)	Critically Low	Serious	N/A	N/A	N/A	N/A	N/A	N/A	N/A	N/A
Wang et al. [44]	MCI	Moderate	Not serious	Serious	Serious	Serious	Not reported	Ignored	Not	Not	Low ⊕ ⊕
Yang et al. [45]	MCI	Critically Low	Serious	N/A	N/A	N/A	N/A	N/A	N/A	N/A	N/A
Malmberg et al. [46]	MCI/Dementia	High	Not serious	Serious	Not serious	Serious	Yes	Medium	Yes	Yes	Low ⊕ ⊕
Jung et al. [47]	MCI/SMC	High	Not serious	Serious	Not serious	Serious	Not	Medium	Yes	Yes	Low ⊕ ⊕
Meng et al. [48]	MCI/mNCD	Critically Low	Serious	Not serious	Not serious	Serious	Not reported	Small	Yes	Not	Low ⊕ ⊕
Ali et al. [49]	MCI/AD	Moderate	Not serious	Not serious	Not serious	Not serious	Not	Small	Yes	Not	Moderate ⊕ ⊕ ⊕
Wu et al. [50]	MCI	Moderate	Not serious	Not serious	Not serious	Not serious	Not	Medium	Yes	Not	Moderate ⊕ ⊕ ⊕
Qing Yi et al. [51]	MCI (amnestic MCI and non-amnestic MCI)	Moderate	Not serious	Serious	Not serious	Not serious	Yes	Medium	Yes	Not	Moderate ⊕ ⊕ ⊕
Ying et al. [52]	MCI	High	Serious	Serious	Not serious	Not serious	Yes	Medium	Yes	Not	High ⊕ ⊕ ⊕ ⊕

*MCI* Mild cognitive impairment, *AD* Alzheimer's Disease, *SMC* Subjective Memory Complaints, *mNCD* mild neurocognitive disorders

Grade: ⊕ ⊕ ⊕ ⊕ = High certainty, ⊕ ⊕ ⊕ = Moderate certainty, ⊕ ⊕ = Low certainty, ⊕ = very low certainty. N/A = Not Applicable; These domains were not assessed in systematic reviews providing exclusively narrative syntheses without pooled quantitative estimates, as standard GRADE domains such as inconsistency, imprecision, and publication bias require quantitative meta-analytic data

Regarding the GRADE evaluation, two outcomes across the sixteen reviews were rated as high certainty, four as moderate, four as low, and six as very low. The primary reasons for downgrading were related to serious or very serious risks of bias, inconsistency, and imprecision, mainly due to heterogeneity in intervention protocols, small sample sizes, and limited statistical power.

Findings were interpreted according to a pre-specified evidence hierarchy. Greater interpretive weight was assigned to outcomes from reviews rated as high or moderate methodological quality (AMSTAR 2) with moderate or high certainty of evidence (GRADE). Multicomponent interventions yielded robust effects on executive function (SMD = 0.35–0.40) [49, 51], and memory (SMD = 0.28–0.30) [49, 51]. Regarding intervention design, the weight of evidence favors simultaneous training over sequential approaches [46], with more pronounced effect sizes for cognition (g = 0.385; *p* < 0.001) compared to sequential designs (g = 0.114; *p* = 0.301) [40]. Conversely, other domains exhibit a weaker strength of inference; for instance, the impact on attention failed to reach statistical significance in the most rigorous analyses (*p* = 0.1) [49]. Furthermore, while positive effects on global cognition were reported in some reviews, the certainty of these findings is deemed very low due to critical limitations involving imprecision, inconsistency, and risk of bias in the primary studies [41].

### Overlap and Clinical trials information

Across the 16 included systematic reviews, 322 randomized controlled trials were reported. Of these, 143 met eligibility criteria (N), resulting in 84 unique primary trials (r) after overlap removal. Overlap analysis demonstrated a slight degree of overlap among the included reviews (CCA = 4.68%; Supplemental Material 6), indicating a relatively low risk of double-counting of primary studies. However, narrative synthesis was retained because of the substantial heterogeneity in intervention components, duration, outcome assessment, and methodological approaches across reviews. The included trials comprised 5,818 participants with mild cognitive impairment, with a median age of 74.8 years (IQR: 71.7–77.6). The median session duration was 60 min (IQR: 45–92.5), with a frequency of two sessions per week (IQR: 2–3) over a median period of 16 weeks (IQR: 10–24), amounting to a total of 36 sessions (IQR: 23–60). Further details of the individual clinical trials are provided in Supplemental Material 7.

The original RCTs evaluated a variety of non-pharmacological interventions, with cognitive training activities being the most frequent (54.7%; 46/84), followed by physical exercise programs (47.6%; 40/84), Wii or computer-based games (21.4%; 18/84), balance training (11.9%; 10/84), Tai Chi (10.7%; 9/84), and lifestyle-based interventions (9.5%; 8/84). Social and recreational activities were reported in 5.9% of studies (5/84), as were dual-task interventions (5.9%; 5/64). Less frequently reported strategies included virtual reality, walking programs, relaxation techniques, nutrition counseling, art-based activities, reminiscence therapy, motivational approaches, multisensory stimulation, group therapy, poetry recitation, and educational interventions. Cognitive training was most frequently combined with physical exercise (20 studies), followed by combinations involving Tai Chi, activities of daily living, computer-based games, and balance training.

**Fig. 2:**
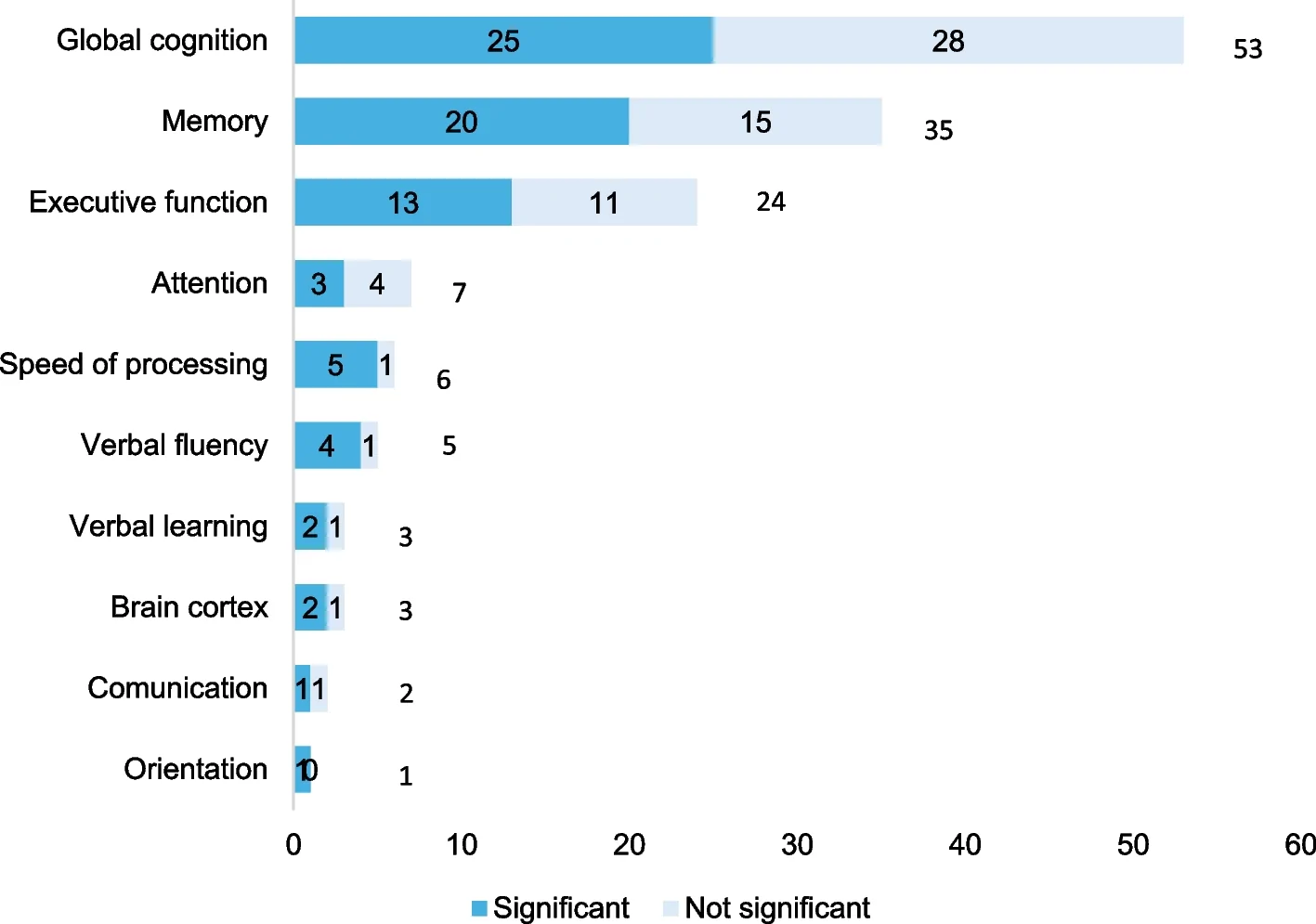
Distribution of positive findings across cognitive domains. The numbers represent the number of RCTs reporting statistically significant and non-significant findings within each cognitive domain and do not reflect pooled effect estimates. RCTs: randomized controlled trials

The most frequently assessed cognitive domains among the 84 randomized controlled trials (RCTs) were global cognition (*n* = 53), memory (*n* = 35), executive function (*n* = 24), attention (*n* = 7), and processing speed (*n* = 6). The least commonly evaluated domains included communication, verbal fluency, verbal learning, orientation, and cortical brain function. Significant positive effects of multicomponent interventions were observed in 57.1% of studies (20/35) assessing memory, 54.1% (13/24) assessing executive function, 47.1% (25/53) assessing global cognition, and 42.9% (3/7) assessing attention. These proportions reflect the frequency of statistically significant findings at the trial level and should not be interpreted as pooled efficacy estimates or indicators of effect magnitude consistency. (see Fig. 2).

These findings suggest that memory and executive function are the cognitive domains most responsive to multicomponent non-pharmacological interventions in older adults with mild cognitive impairment.

## Discussion

This umbrella review synthesized evidence from sixteen systematic reviews, comprising five systematic reviews without meta-analysis and eleven systematic reviews with meta-analyses, encompassing 322 randomized controlled trials, of which 143 met eligibility criteria, yielding 84 unique primary trials after overlap removal (CCA = 4.68%), comprising 5,818 participants, evaluating the effects of multicomponent non-pharmacological interventions in older adults with mild cognitive impairment (MCI). Overall, the findings suggest potential cognitive benefits under structured intervention conditions, particularly in memory, executive function, and global cognition [37–40, 42–44]. These results indicate that multidomain programs combining cognitive and physical activities may contribute to improvements in cognitive performance and functional independence [32–45]. However, these findings should be interpreted cautiously given the variability in methodological quality (AMSTAR 2) and certainty of evidence (GRADE).

Importantly, the strength of evidence varied considerably across cognitive domains and closely aligned with AMSTAR-2 confidence ratings. Outcomes related to memory and executive function were more frequently supported by reviews rated as high or moderate quality, lending greater confidence to these effects. In contrast, evidence for global cognition and other domains was mainly derived from reviews assessed as low or critically low quality, suggesting that these findings should be interpreted with caution.

Several included reviews incorporated mixed diagnostic populations (e.g., MCI with dementia or subjective cognitive complaints). Although MCI-specific data were extracted where available, the inclusion of mixed samples may introduce indirectness and should be considered when interpreting pooled trends.

Despite the overall consistency of favorable trends, the magnitude of observed effects remained modest [49]. Across the quantitative meta-analyses reporting standardized effect estimates, SMDs or Hedges’ g values generally ranged from approximately 0.20 to 0.40 for global cognition, memory, and executive function, with higher values observed in selected domains or intervention formats. These estimates were reported mainly in meta-analyses evaluating combined physical–cognitive or dual-task interventions [40, 46, 48, 49, 51]. However, frequent downgrades in certainty due to risk of bias, inconsistency, and imprecision limit the strength of causal inference and preclude broad generalizations.

Since approximately 2016, there has been a growing number of studies evaluating multicomponent interventions combining cognitive stimulation with structured physical activity. These combinations seem particularly relevant for domains supported by higher-quality evidence, such as memory and executive function, suggesting potential synergistic effects [46]. However, the substantial heterogeneity in intervention composition, intensity, duration, and delivery modality precludes identification of an optimal intervention dosage and limits direct comparability across programs.

When prioritizing evidence based on methodological rigor, the effectiveness of multicomponent interventions appears non-uniform across cognitive domains, with the most robust impact and highest certainty localized in memory and executive functions. This selectivity suggests that cognitive control processes and information storage are particularly sensitive to the synergy of combined physical-cognitive training, especially within simultaneous modalities. Conversely, the pronounced uncertainty observed in global cognition and attention frequently attributed to methodological limitations and risk of bias in lower-quality reviews highlights the need for cautious clinical interpretation and further research employing standardized experimental designs.

Across trials, a recurring intervention pattern emerged, clustering around 12–24 weeks with biweekly sessions of approximately 60 min. Although insufficient to define an optimal dosage, this convergence suggests a plausible intervention window warranting prospective evaluation.

Outcomes related to attention, language, communication, and broader aspects of global cognition remain underrepresented in higher-quality reviews. The current evidence base for these domains is characterized by greater heterogeneity and lower confidence, highlighting important conceptual and methodological gaps that limit clinical applicability and comprehensive assessment of cognitive health in MCI.

Acceptability and adherence are critical factors influencing the long-term success of these interventions. Available evidence suggests that multicomponent programs are as acceptable as single-domain approaches [53], but the relative contribution of individual components to long-term engagement remains insufficiently understood.

Although sleep disturbances, caregiver burden, and digital caregiver interventions were not directly evaluated outcomes within the included systematic reviews and meta-analyses, these factors have been identified in the broader geriatric and dementia-related literature as clinically relevant components that may influence cognitive health, intervention adherence, and quality of life in older adults with mild cognitive impairment [54, 55]. In particular, sleep disturbances such as insomnia have been associated with cognitive and functional impairment and may act as potential moderating factors when interpreting intervention outcomes rather than primary targets of the reviewed interventions [54].

Likewise, external evidence suggests that multicomponent and digitally supported caregiver interventions may contribute to reductions in caregiver distress and improvements in self-efficacy, which could indirectly influence patient-related outcomes [14, 56]. However, these observations derive from literature beyond the scope of the present umbrella review and should therefore be interpreted as broader contextual considerations rather than findings directly supported by the included evidence synthesis.

The main strengths of this umbrella review include the systematic synthesis of evidence from SRMAs and the detailed characterization of intervention frequency, duration, and session structure. This level of detail provides a clearer understanding of the relationship between intervention design and cognitive outcomes, particularly in domains where effects were most consistently supported by higher-quality evidence.

The main limitation for inference was the variable methodological quality of the included reviews. A substantial proportion were rated as low or critically low using AMSTAR 2, primarily due to absent or incomplete protocol registration, failure to report excluded studies, inadequate integration of risk-of-bias findings into conclusions, and lack of funding source disclosure. In addition, several included systematic reviews provided narrative syntheses without pooled quantitative estimates, limiting the applicability of standard GRADE domains such as inconsistency, imprecision, and publication bias. Consequently, certainty assessments for these reviews could not be fully standardized, which may have affected comparability across evidence profiles. Although overlap among reviews was low according to the CCA estimate, substantial clinical and methodological heterogeneity across intervention characteristics, outcome measures, follow-up periods, and synthesis approaches limited the feasibility and interpretability of an additional quantitative synthesis at the umbrella review level.

To address these limitations, a hierarchical interpretation of the evidence was conducted, prioritizing findings from reviews with higher methodological rigor and certainty according to GRADE criteria. This analytical approach ensured that the conclusions derived from this umbrella review were not based on a simple vote-counting method, but rather on the strength and consistency of the available evidence. Consistent findings for memory and executive function were supported by higher-quality reviews, strengthening confidence in these domains despite the underlying heterogeneity across interventions and study designs. An additional source of indirectness relates to the inclusion of some systematic reviews enrolling mixed populations with both MCI and dementia. Although only reviews allowing extraction or interpretation of MCI-specific findings were included, inconsistent reporting of subgroup characteristics across reviews may have limited the precision of population-specific inferences.

Finally, ongoing research into comprehensive, multicomponent care strategies for older adults is essential to preserve cognitive and functional capacity. Future studies should adopt robust, preregistered protocols, standardized cognitive assessments, and longer follow-up periods, considering comorbidities that require integrated, context-sensitive management approaches [55, 57]. The predominant focus on global cognition, memory, and executive function reflects a limited conceptualization of cognitive health in MCI, while the underassessment of communication, verbal fluency, and higher cortical functions restricts clinical applicability. Addressing these gaps, along with the context-sensitive management of comorbidities, will enhance the translational relevance of multicomponent interventions [58]. The findings of this umbrella review have implications for diverse stakeholders in the management of MCI. For healthcare providers, the evidence supports the potential benefit of synergistic, multicomponent protocols especially those integrating physical and cognitive stimulation as a primary non-pharmacological strategy. For policymakers, the breadth of the 84 unique primary studies synthesized here suggests the potential feasibility of these interventions, indicating they could be considered for integration into public health programs, although further high-quality evidence is required before large-scale implementation. Finally, for patients and caregivers, these findings suggest potential cognitive benefits under structured conditions.

## Conclusion

This umbrella review included sixteen studies (five meta-analyses and eleven systematic reviews) encompassing 84 unique randomized controlled trials (RCTs) and 5,818 participants with mild cognitive impairment (MCI), all of whom received non-pharmacological multicomponent interventions. Overall, the findings suggest potential cognitive benefits, particularly in memory and executive function, though the certainty of these findings varied across reviews. Conversely, improvements in global cognition were largely observed in lower-quality reviews and should therefore be interpreted with considerable caution.

Although favorable effects were frequently observed, their magnitude remained modest, underscoring the need for further research to optimize intervention design and enhance the durability of cognitive benefits. Multicomponent interventions combining cognitive stimulation with structured physical activity were the most frequently studied approach in recent trials; however, whether this combination confers differential benefits over other configurations remains uncertain given current evidence. However, the heterogeneity in intervention components, intensity, and duration limits definitive conclusions regarding their comparative effectiveness.

Future research should prioritize methodologically rigorous, preregistered studies with standardized outcome frameworks, longer follow-up periods, and clearer characterization of intervention components. Whether the incorporation of context-sensitive strategies addressing comorbidities such as sleep disturbances, or the inclusion of caregiver-related outcomes, would enhance effectiveness or implementation feasibility remains to be examined. Given global population aging and the growing burden of MCI, advancing high-quality research on multicomponent interventions remains necessary to determine whether multicomponent interventions can yield scalable and reproducible benefits in both clinical and community settings.

## Supplementary Information


Supplementary Material 1.


## Data Availability

No datasets were generated or analysed during the current study.
