# Supplementary material for: Non-pharmacological interventions for older adults with mild cognitive impairment: an umbrella review
Source: BMC Geriatr. 2026 Aug 13;26:1103. doi: 10.1186/s12877-026-07933-6 (PMC13523256; doi:10.1186/s12877-026-07933-6)
Supplement: Supplementary file 1 [file 12877_2026_7933_MOESM1_ESM.pdf]

Supplemental material 1 PRIOR Checklist

(Gates M, Gates A, Pieper D, et al. Reporting guideline for overviews of reviews of healthcare interventions: development of the PRIOR statement. *BMJ* 2022;378:e070849. doi:10.1136/bmj-2022-070849.)

**Section** Topic	#	**Item**	**Location reported**
**TITLE**			page
Title	1	Identify the report as an overview of reviews.	1
**ABSTRACT**			
Abstract	2	Provide a comprehensive and accurate summary of the purpose, methods, and results of the overview of reviews.	2 and 3
**INTRODUCTION**			
Rationale	3	Describe the rationale for conducting the overview of reviews in the context of existing knowledge.	4-6
Objectives	4	Provide an explicit statement of the objective(s) or question(s) addressed by the overview of reviews.	6
**METHODS**			
Eligibility criteria	5a	Specify the inclusion and exclusion criteria for the overview of reviews. If supplemental primary studies were included, this should be stated, with a rationale.	8
	5b	Specify the definition of 'systematic review' as used in the inclusion criteria for the overview of reviews.	7
Information sources	6	Specify all databases, registers, websites, organizations, reference lists, and other sources searched or consulted to identify systematic reviews and supplemental primary studies (if included). Specify the date when each source was last searched or consulted.	7
Search strategy	7	Present the full search strategies for all databases, registers and websites, such that they could be reproduced. Describe any search filters and limits applied.	7, 8 Supp 3
Selection process	8a	Describe the methods used to decide whether a systematic review or supplemental primary study (if included) met the inclusion criteria of the overview of reviews.	9
	8b	Describe how overlap in the populations, interventions, comparators, and/or outcomes of systematic reviews was identified and managed during study selection.	11
Data collection process	9a	Describe the methods used to collect data from reports.	10
	9b	If applicable, describe the methods used to identify and manage primary study overlap at the level of the comparison and outcome during data collection. For each outcome, specify the method used to illustrate and/or quantify the degree of primary study overlap across systematic reviews.	11
	9c	If applicable, specify the methods used to manage discrepant data across systematic reviews during data collection.	9
Data items	10	List and define all variables and outcomes for which data were sought. Describe any assumptions made and/or measures taken to identify and clarify missing or unclear information.	9
Risk of bias assessment	11a	Describe the methods used to *assess* risk of bias or methodological quality of the included systematic reviews.	10
	11b	Describe the methods used to *collect* data on (from the systematic reviews) and/or *assess* the risk of bias of the primary studies included in the systematic reviews. Provide a justification for instances where flawed, incomplete, or missing assessments are identified but not re-assessed.	10
	11c	Describe the methods used to *assess* the risk of bias of supplemental primary studies (if included).	N/A
Synthesis methods	12a	Describe the methods used to summarize or synthesize results and provide a rationale for the choice(s).	10-11
	12b	Describe any methods used to explore possible causes of heterogeneity among results.	N/A
	12c	Describe any sensitivity analyses conducted to assess the robustness of the synthesized results.	N/A
Reporting bias assessment	13	Describe the methods used to *collect* data on (from the systematic reviews) and/or *assess* the risk of bias due to missing results in a summary or synthesis (arising from reporting biases at the levels of the systematic reviews, primary studies, and supplemental primary studies, if included).	N/A
Certainty assessment	14	Describe the methods used to *collect* data on (from the systematic reviews) and/or *assess* certainty (or confidence) in the body of evidence for an outcome.	N/A
**RESULTS**			
Systematic review and supplemental primary study selection	15a	Describe the results of the search and selection process, including the number of records screened, assessed for eligibility, and included in the overview of reviews, ideally with a flow diagram.	12-13
	15b	Provide a list of studies that might appear to meet the inclusion criteria, but were excluded, with the main reason for exclusion.	Supp 4

**Section** Topic	**#**	**Item**	**Location reported**
Characteristics of systematic reviews and supplemental primary studies	16	Cite each included systematic review and supplemental primary study (if included) and present its characteristics.	16
Primary study overlap	17	Describe the extent of primary study overlap across the included systematic reviews.	14 -15
Risk of bias in systematic reviews, primary studies, and supplemental primary studies	18a	Present assessments of risk of bias or methodological quality for each included systematic review.	23 -24
	18b	Present assessments (*collected* from systematic reviews or *assessed* anew) of the risk of bias of the primary studies included in the systematic reviews.	N/A
	18c	Present assessments of the risk of bias of supplemental primary studies (if included).	N/A
Summary or synthesis of results	19a	For all outcomes, summarize the evidence from the systematic reviews and supplemental primary studies (if included). If meta-analyses were done, present for each the summary estimate and its precision and measures of statistical heterogeneity. If comparing groups, describe the direction of the effect.	17-22
	19b	If meta-analyses were done, present results of all investigations of possible causes of heterogeneity.	N/A
	19c	If meta-analyses were done, present results of all sensitivity analyses conducted to assess the robustness of synthesized results.	N/A
Reporting biases	20	Present assessments (*collected* from systematic reviews and/or *assessed* anew) of the risk of bias due to missing primary studies, analyses, or results in a summary or synthesis (arising from reporting biases at the levels of the systematic reviews, primary studies, and supplemental primary studies, if included) for each summary or synthesis assessed.	23-24
Certainty of evidence	21	Present assessments (*collected* or *assessed* anew) of certainty (or confidence) in the body of evidence for each outcome.	23-24
**DISCUSSION**			
Discussion	22a	Summarize the main findings, including any discrepancies in findings across the included systematic reviews and supplemental primary studies (if included).	27
	22b	Provide a general interpretation of the results in the context of other evidence.	28-29
	22c	Discuss any limitations of the evidence from systematic reviews, their primary studies, and supplemental primary studies (if included) included in the overview of reviews. Discuss any limitations of the overview of reviews methods used.	29-30
	22d	Discuss implications for practice, policy, and future research (both systematic reviews and primary research). Consider the relevance of the findings to the end users of the overview of reviews, e.g., healthcare providers, policymakers, patients, among others.	29 30
**OTHER INFORMATION**			
Registration and protocol	23a	Provide registration information for the overview of reviews, including register name and registration number, or state that the overview of reviews was not registered.	7
	23b	Indicate where the overview of reviews protocol can be accessed, or state that a protocol was not prepared.	7
	23c	Describe and explain any amendments to information provided at registration or in the protocol. Indicate the stage of the overview of reviews at which amendments were made.	N/A
Support	24	Describe sources of financial or non-financial support for the overview of reviews, and the role of the funders or sponsors in the overview of reviews.	In plataform
Competing interests	25	Declare any competing interests of the overview of reviews' authors.	In plataform
Author information	26a	Provide contact information for the corresponding author.	1
	26b	Describe the contributions of individual authors and identify the guarantor of the overview of reviews.	In plataform

Availability of data and other materials	27	Report which of the following are available, where they can be found, and under which conditions they may be accessed: template data collection forms; data collected from included systematic reviews and supplemental primary studies; analytic code; any other materials used in the overview of reviews.	Supplemental material

Supplemental Material 2. Search strategy (Pubmed)

(Cognitive Dysfunction[Title]) OR (Cognitive Decline[Title]) OR (Cognitive Declines[Title]) OR (Cognitive Dysfunctions[Title]) OR (Cognitive Impairment[Title]) OR (Cognitive Impairment, Mild[Title]) OR (Cognitive Impairments[Title]) OR (Cognitive Impairments, Mild[Title]) OR (Decline, Cognitive[Title]) OR (Declines, Cognitive[Title]) OR (Deterioration, Mental[Title]) OR (Deteriorations, Mental[Title]) OR (Disorder, Mild Neurocognitive[Title]) OR (Disorders, Mild Neurocognitive[Title]) OR (Dysfunction, Cognitive[Title]) OR (Dysfunctions, Cognitive[Title]) OR (Impairment, Cognitive[Title]) OR (Impairment, Mild Cognitive[Title]) OR (Impairments, Cognitive[Title]) OR (Impairments, Mild Cognitive[Title]) OR (Mental Deterioration[Title]) OR (Mental Deteriorations[Title]) OR (Mild Cognitive Impairment[Title]) OR (Mild Cognitive Impairments[Title]) OR (Mild Neurocognitive Disorder[Title]) OR (Mild Neurocognitive Disorders[Title]) OR (Neurocognitive Disorder, Mild[Title]) OR (Neurocognitive Disorders, Mild[Title]) OR (cognitive[Title]) OR (neurocognitive[Title]) OR (cognitive performance[Title]) AND (((Efficacy[Title/Abstract] OR effect[Title/Abstract] OR effects[Title/Abstract] OR improvement[Title/Abstract] OR effectiveness[Title/Abstract]) AND (intervention[Title/Abstract] OR Interventions[Title/Abstract] OR Program[Title/Abstract] OR programs[Title/Abstract] OR nonpharmacological[Title/Abstract] OR Training[Title/Abstract] OR rehabilitation[Title/Abstract])) AND (Elderly[Title/Abstract] OR Old[Title/Abstract] OR Older[Title/Abstract] OR Ancient[Title/Abstract] OR aged[Title/Abstract])) AND (Review[Title/Abstract] OR Reviews[Title/Abstract] OR "Sistematic review"[Title/Abstract] OR "Review Literature"[Title/Abstract] OR "State of the Art"[Title/Abstract] OR Meta-Analysis[Title/Abstract])Supplemental Material 3. Research question according to the PICO framework

Supplemental Material 3. PICO question

Population	*Included*<br>community-dwelling older adults (over 60 years) with mild cognitive impairment.<br>*Excluded*<br>Healthy people, people with dementia or Alzheimer's
Intervention	*Included*<br>Non-pharmacological and multicomponent interventions related to lifestyles, such as: A combination of interventions in diet and nutrition, physical activity and exercise, psychological, cognitive training or rehabilitation, stress management, and complementary or alternative therapies.<br>*Excluded*<br>Exclusions: Scoping reviews, narrative reviews, systematic reviews of non-experimental studies, animal studies, in vitro studies, studies evaluating hormone replacement, supplementation of micronutrients such as vitamins, minerals, amino acids, or essential and non-essential fatty acids, feeding formulas will be excluded, specialized, protein powder, or other presentations. Editorials, review protocols with no results, reviews including wave stimulation interventions (electrical and ultrasound), reviews of a single component study, studies in which changes in cognition were not one of the primary outcomes, and studies in healthy people, hospitalized or dementia patients.
Comparator	*Included*<br>Standard care, usual or single intervention, or no intervention, or placebo, or active/passive control.
Outcomes	Changes in cognitive function evaluated through assessment by a psychiatrist, neurologist, or any evaluation test, such as the Mini-Mental State Examination MMSE, the MoCa test, the Mini-Cog, and the Foto-Test, among others.

Supplemental Material 4. Excluded full-text articles and reasons

Year	Authors	Title	Reason for final exclusion
2013	Lin JS, O'Connor E, Rossom RC, Perdue LA, Burda BU, Thompson M, and Eckstrom E.	Screening for Cognitive Impairment in Older Adults: An Evidence Update for the U.S. Preventive Services Task Force [Internet]	See results on p. 62, p. 163, p. 355. One of the two articles reviewed interventions that include medications.
2019	Sanders LMJ, Hortobágyi T, la Bastide-van Gemert S, van der Zee EA, and van Heuvelen MJG	Dose-response relationship between exercise and cognitive function in older adults with and without cognitive impairment: A systematic review and meta-analysis.	Only 6 articles out of 36 were included because the others were in healthy people or were single-component interventions.
2020	Sheng C, Yang K, Wang X, Li H, Li T, Lin L, Liu Y, Yang Q, Wang X, Wang X, Sun Y, and Han Y.	Advances in Non-Pharmacological Interventions for Subjective Cognitive Decline: A Systematic Review and Meta-Analysis.	It deals with subjective cognitive decline (SCD).
2019	Liang JH, Lin L, Wang YQ, Jia RX, Qu XY, Li J, Li JY, Qian S, Qian YX, Wang S, Gao Z, Cheng XX, and Xu Y.	Non-pharmacological therapeutic strategy options for patients with dementia based on cognitive function-A Bayesian network meta-analysis of randomized controlled trials.	Transdisciplinary multicomponent is not met
2021	Zhang X, Liu Y, Zhang, W, Pan N, Cherkashin E, Kruszewski A, and Cherkashin I.	The effect of Chinese traditional exercise on cognitive function improvement in the elderly - meta-analysis	It does not include multidomain interventions (only traditional Chinese physical exercises).
2014	Law LL, Barnett F, Yau MK, and Gray MA.	Effects of combined cognitive and exercise interventions on cognition in older adults with and without cognitive impairment: A systematic review	Of 5 possible articles, 2 are non-RCTs and 1 addresses cognitive complaints; there is no separate analysis of these.
2013	Gates N, Fiatarone Singh MA, Sachdev PS, and Valenzuela M.	The effect of exercise training on cognitive function in older adults with mild cognitive impairment: A meta-analysis of randomized controlled trials	Of the 4 possible articles, the multimodal aspect of the interventions is the physical dimension.
2019	Lampit A, Hallock H, and Valenzuela M.	Dissecting the anatomy of computerized cognitive training: Systematic review and meta-analysis of RCTs in older adults	Congress abstract
2013	Reijnders J, van Heugten C, and van Boxtel M.	Cognitive interventions in healthy older adults and people with mild cognitive impairment: A systematic review	Of the 6 articles on cognitive impairment, only 1 addresses a multicomponent intervention.

Supplemental Material 5. AMSTAR 2

Q1	Did the research questions and inclusion criteria for the review include the components of PICO?
Q2	Did the report of the review contain an explicit statement that the review methods were established prior to the conduct of the review and did the report justify any significant deviations from the protocol?
Q3	Did the review authors explain their selection of the study designs for inclusion in the review?
Q4	Did the review authors use a comprehensive literature search strategy?
Q5	Did the review authors perform study selection in duplicate?
Q6	Did the review authors perform data extraction in duplicate?
Q7	Did the review authors provide a list of excluded studies and justify the exclusions?
Q8	Did the review authors describe the included studies in adequate detail?
Q9	Did the review authors use a satisfactory technique for assessing the risk of bias (RoB) in individual studies that were included in the review?
Q10	Did the review authors report on the sources of funding for the studies included in the review?
Q11	If meta-analysis was performed did the review authors use appropriate methods for statistical combination of results?
Q12	If meta-analysis was performed, did the review authors assess the potential impact of RoB in individual studies on the results of the meta-analysis or other evidence synthesis?
Q13	Did the review authors account for RoB in individual studies when interpreting/discussing the results of the review?
Q14	Did the review authors provide a satisfactory explanation for, and discussion of, any heterogeneity observed in the results of the review?
Q15	If they performed quantitative synthesis did the review authors carry out an adequate investigation of publication bias (small study bias) and discuss its likely impact on the results of the review?
Q16	Did the review authors report any potential sources of conflict of interest, including any funding they received for conducting the review?

Supplemental Material 6. Overlap analysis

RCT author, year	Ali et al.(49)	Carrion,(39)	Gates,(41)	Gheysen,(40)	Guo, (43)	Jung,(47)	Kallio,(38)	Malmberg,(46)	Meng , (48)	Öhman, (37)	Özbe , (42)	QingYi, (51)	Wang, (44)	Wu, (50)	Yang,(45)	Ying , (52)	Total
Amjad, 2019									X								**1**
Anderson-, Hanley, 2018						X		X	X						X		**4**
Bae, 2019	X				X									X		X	**4**
Baglio, 2015		X									X						**2**
Barban, 2016							X										**1**
Barcelos, 2015								X									**1**
Barnes, 2013			X	X													**2**
Bottino, 2005							X										**1**
BS, Silva, 2017	X																**1**
BS, Silva, 2018	X																**1**
Burgener, 2008										X							**1**
Buschert, 2011											X					X	**2**
Chen, 2018	X																**1**
Combourieu, Donnezan, 2018								X	X						X		**3**
Cott, 2002										X							**1**
Damirchi, 2017														X			**1**
Damirchi, 2018								X				X					**2**
de Souto Barreto, 2021						X											**1**
Delbroek, 2017	X							X				X			X	X	**5**
Doi, 2017													X				**1**
Donnezan, 2018	X													X		X	**3**
Fernández-Calvo, 2015							X										**1**
Fiatarone Singh, 2014			X					X	X						X		**4**
Fogarty, 2016															X		**1**
Friedman and Tappen, 1991										X							**1**
Gill, 2016	X																**1**
Gooding, 2016			X														**1**
Graessel, 2011											X						**1**
Griffiths, 2020																X	**1**
Guo, 2013													X				**1**
Hagovska, 2016				X	X			X									**3**
Hagovska, 2017	X								X						X		**3**
Heath, 2016	X																**1**
Hughes, 2014				X				X	X								**3**
Jeong, 2021																X	**1**
Jiranan, 2020									X								**1**
Joke, 2017									X								**1**
Karssemeijer,2019								X									**1**
Kim, 2016		X															**1**
Köbe, 2016									X								**1**
Kounti, 2011												X				X	**2**

Kurz, 2009																X	**1**
Lam, 2012				X	X												**2**
Lam, 2015									X				X		X	X	**4**
Law, 2019									X								**1**
Lemke, 2018	X																**1**
Li, 2019						X											**1**
Liao, 2019						X						X					**2**
Liao, 2020												X					**1**
Lipardo, 2020,												X					**1**
Low, 2014		X															**1**
Luttenberger, 2012		X															**1**
Maci, 2012											X						**1**
Maci, 2015							X										**1**
Maffei, 2017	X							X							X	X	**4**
Makizako, 2012	X																**1**
Montero-Odasso, 2023																X	**1**
Mrakic-Sposta, 2018					X			X									**2**
Muñiz, 2015, [51]		X															**1**
Ngandu, 2015		X															**1**
Obman, 2016	X																**1**
Olazaran, 2004							X				X						**2**
Park, 2019	X				X			X	X			2				X	**7**
Phirom2020, 9						X											**1**
Poptsi, 2022,												X					**1**
Rapp, 2002											X					X	**2**
Rojas, 2013																X	**1**
Schwenk, 2010											X					X	**2**
Schwenk, 2016									X								**1**
Shimada, 2017	X								X								**2**
Shimada, 2018								X				X			X	X	**4**
Singh, 2014					X												**1**
Sungkarat, 2017				X	X												**2**
Suzuki, 2012	X											X		X			**3**
Suzuki, 2013	X			X					X	X	X	X	X				**7**
Troyer, 2008											X						**1**
Tsolaki, 2011																X	**1**
Ventrolli, 2016	X																**1**
Venturelli, 2016		X															**1**
Wiloth, 2018	X																**1**
Wing Ching, 2016	X																**1**
Xu, 2020																X	**1**
Yang, 2022																X	**1**
Zijun, 2020									X								**1**
**Total**	**20**	**7**	**3**	**6**	**7**	**5**	**5**	**13**	**16**	**4**	**9**	**12**	**4**	**4**	**9**	**19**	**143**

Supplemental Material 7. Research characteristics

Author	Year	n	Average age	Session				Dominion										Intervention																	
				Session duration min.	Frequency per week	Treatment duration (weeks)	Total number of sessions	Global cognition	Executive function	Attention	Memory	Communication	Speed of processing	Verbal fluency	Verbal learning	Orientation	Brain cortex	Physical exercise	Cognitive activity	Nutritional	Social activities/recreation	Art	Lifestyle-based	Wii games/Computerized	Virtual reality	Tai Chi	Reminiscence therapy	Motivational therapeutic	Dual-task	Balance training	Multidimensional stimulation group therapy	Relaxation skill	Walk	Reciting poems	Educational program
Venturelli	2016	80	85	60	5	12	60	0	.	.	.	.	.	.	.	.	.	1	1	0	0	0	0	0	0	0	0	0	0	0	0	0	0	0	0
Kim	2016	53	78	60	5	24	120	0	0	0	.	.	.	.	.	.	.	0	0	0	0	1	0	0	0	0	0	0	0	0	0	0	0	0	0
Law	2014	83	72.8	60	1	10	13	1	.	.	.	.	.	.	1	.	.	1	0	0	0	0	0	0	0	0	0	0	0	0	0	0	0	0	0
Luttenberger	2012	61	84.3	120	6	48	288	0	.	.	.	.	.	.	.	.	.	1	1	0	0	0	0	0	0	0	0	0	0	0	0	0	0	0	0
Muñiz	2015	84	74.9	210	2	.	.	0	.	.	.	.	.	.	.	.	.	1	1	0	0	0	0	0	0	0	0	0	0	0	0	0	0	0	0
Ngandu	2015	1.260	69.5	.	.	96	.	1	1	.	1	.	1	.	.	.	.	0	0	0	0	0	1	0	0	0	0	0	0	0	0	0	0	0	0
Barcelos	2015	17	85.1	45	5	12	60	.	.	.	.	.	.	.	.	.	.	1	0	0	0	0	0	1	0	0	0	0	0	0	0	0	0	0	0
Hughes	2014	20	77.4	90	1	24	24	0	.	.	.	.	.	.	.	.	.	0	1	0	0	0	0	1	0	0	0	0	0	0	0	0	0	0	0
Karssemeijer	2019	101	79.2	50	3	12	36	.	0	.		.	.	.	.	.	.	1	1	0	0	0	0	0	0	0	0	0	0	0	0	0	0	0	0
Mrakic-Sposta	2018	10	73.5	45	3	6	18	0	.	.	.	.	.	.	.	.	.	0	0	0	0	0	0	0	1	0	0	0	0	0	0	0	0	0	0
Lam	2012	389	77.2	30	3	48	144	0	0	.	.	.	.	0	.	.	.	0	1	0	0	0	0	0	0	1	0	0	0	0	0	0	0	0	0
Sungkarat	2017	66	68.3	50	3	15	45	0	1	.	1	.	.	.	.	.	.	0	0	0	0	0	0	0	0	1	0	0	0	0	0	0	0	0	0
Siu	2018	160	.	60	2	16	32	1	.	.	.	.	.	.	.	.	.	0	0	0	0	0	0	0	0	1	0	0	0	0	0	0	0	0	0
Fiatarone	2014	49	70.1	100	3	24	72	0	1	1	1	.	.	.	.	.	.	0	0	0	0	0	0	0	0	1	0	0	0	0	0	0	0	0	0
Damirchi	2018	44	67.76	60	3	8	24	0	.	.	1	.	1	.	.	.	.	0	0	0	0	0	0	0	0	0	0	0	0	0	0	0	0	0	0
Singh	2014	86	79.1	100	2	24	48	0	.	.	.	.	.	.	.	.	.	1	1	0	0	0	0	0	0	0	0	0	0	0	0	0	0	0	0
Bae	2019	83	75.9	90	2	24	48	0	0	.	1	.	.	.	.	.	.	1	1	0	1	0	0	0	0	0	0	0	0	0	0	0	0	0	0
Bottino	2005	13	74	90	1	20	20	1	0	.	1	.	.	.	0	.	.	0	1	0	0	0	1	0	0	0	0	0	0	0	0	0	0	0	0
Fernández-Calvo	2015	61	73	40	3	16	48	1	.	.	.	.	.	.	.	.	.	0	1	0	1	0	0	0	0	0	0	0	0	0	0	0	0	0	0
Barban	2016	81	77	30	2	12	24	1	0	.	.	.	.	.	.	.	.	0	1	0	0	0	0	0	0	0	1	0	0	0	0	0	0	0	0
Barnes	2013	126	75	60	3	12	36	1	1	.	1	.	.	1	.	.	.	1	0	0	0	0	0	1	0	0	0	0	0	0	0	0	0	0	0
Gooding	2016	96	75.79	60	2	16	32	1	1	.	1	.	.	.	1	.	.	0	0	0	0	0	0	1	0	0	0	1	0	0	0	0	0	0	0
Optale	2010	36	79	30	3	24	72	1	1	.	1	.	.	1	.	.	.	0	0	0	0	0	0	0	1	0	0	0	0	0	0	0	0	0	0
Suzuki	2013	100	74.8	90	2	24	40	1	.	.	1	.	.	.	.	.	.	0	0	0	0	0	0	0	0	0	0	0	1	0	0	0	0	0	0
Guo	2013	81	74.12	.	.	24	.	.	.	.	.	.	.	.	.	.	.	1	1	0	0	0	0	0	0	0	0	0	0	0	0	0	0	0	0
Hagovská	2016	80	68	30	2	10	20	0	.	.	.	.	.	.	.	.	.	0	1	0	0	0	0	0	0	0	0	0	0	0	0	0	0	0	0
Combourieu Donnezan	2018	69	76.7	60	2	12	12	.	1	.	.	.	.	.	.	.	.	0	0	0	0	0	0	0	0	0	0	0	0	1	0	0	0	0	0
Delbroek	2017	20	87.2	30	2	6	12	0	.	.	.	.	.	.	.	.	.	0	1	0	0	0	0	0	0	0	0	0	1	0	0	0	0	0	0
Fogarty	2016	40		90	2	10	20	.	0	1	1	.	1	.	.	.	.	0	1	0	0	0	0	0	0	1	0	0	0	0	0	0	0	0	0
Hagovska	2017	80	67.7	30	2	10	20	1	.	1	1	.	1	.	.	1	.	1	0	0	0	0	1	0	0	0	0	0	0	0	0	0	0	0	0
Lam	2015	555	76.3	60	3	32	96	0	1	0	1	.	.	.	.	.	.	1	1	0	0	0	0	0	0	0	0	0	0	0	0	0	0	0	0
Maffei	2017	113	74.5	120	6	28	28	1	.	.	.	.	.	.	.	.	0	1	0	0	0	0	0	1	0	0	0	0	0	0	0	0	0	0	0
Shimada	2018	308	71.6	90	1	40	40	1	0	.	0	.	.	.	.	.	1	1	0	0	0	0	0	0	0	0	0	0	1	0	0	0	0	0	0
Baglio	2015	60	75.6	150	3	10	30	0	.	.	.	.	.	.	.	.	.	0	0	0	0	0	0	0	0	0	0	0	0	0	1	0	0	0	0
Buschert	2011	39	71.8	120	1	20	20	1	.	.	0	.	.	.	.	.	.	1	1	0	0	0	0	0	0	0	0	0	0	0	0	0	0	0	0

Author	Year	n	Average age	Session				Dominion										Intervention																	
				Session duration min.	Frequency per week	Treatment duration (weeks)	Total number of sessions	Global cognition	Executive function	Attention	Memory	Communication	Speed of processing	Verbal fluency	Verbal learning	Orientation	Brain cortex	Physical exercise	Cognitive activity	Nutritional	Social activities/recreation	Art	Lifestyle-based	Wii games/Computerized	Virtual reality	Tai Chi	Reminiscence therapy	Motivational therapeutic	Dual-task	Balance training	Multidimensional stimulation group therapy	Relaxation skill	Walk	Reciting poems	Educational program
Graessel	2011	96	84.5	120	6	48	288	1	.	.	.	.	.	.	.	.	.	1	1	0	0	0	1	0	0	0	0	0	0	0	0	0	0	0	0
Maci	2012	14	75	240	5	12	60	0	.	.	.	.	.	.	.	.	.	1	1	0	0	0	0	0	0	0	0	0	0	1	0	0	0	0	0
Olazaran	2004	84	75.3	210	2	58	103	0	.	.	.	.	.	.	.	.	.	1	1	0	0	0	1	0	0	0	0	0	0	0	0	0	0	0	0
Rapp	2002	19	73.3	120	1	6	6	.	.	.	0	.	.	.	.	.	.	0	1	0	0	0	0	0	0	0	0	0	0	0	0	1	0	0	0
Schwenk	2010	61	80.4	120	2	12	24	.	.	.	0	.	.	.	.	.	.	0	0	0	0	0	0	0	0	0	0	0	1	1	0	0	0	0	0
Troyer	2008	48	76	120	1	26	26	.	.	.	1	.	.	.	.	.	.	0	1	0	0	0	0	0	0	0	0	0	0	0	0	1	0	0	0
Lemke	2018	105	82.7	90	2	10	20	.	.	.	.	.	.	.	.	.	.	1	0	0	0	0	0	0	0	0	0	0	0	0	0	0	0	0	0
BS Silva	2017	127	67.7	60	3	24	72	.	.	.	0	.	.	.	.	.	.	1	0	0	0	0	0	1	0	0	0	0	0	0	0	0	0	0	0
Wiloth	2018	99	82.9	90	2	10	20	.	0	.	.	.	.	.	.	.	.	0	1	0	0	0	0	1	0	0	0	0	0	0	0	0	1	0	0
Park	2019	49	71.5	120	.	24	110	0	0	0	0	.	.	.	.	.	.	1	1	0	0	0	0	1	0	0	0	0	0	0	0	0	0	0	0
Bae	2020	280	76.5	90	1	40	40	.	.	.	1	.	.	.	.	.	.	1	0	0	0	0	0	1	0	0	0	0	0	1	0	0	0	1	0
de Souto Barreto	2021	120	75.2	.	2	24	48	0	.	.	.	.	.	.	.	.	.	1	1	1	0	0	0	0	0	0	0	0	0	0	0	0	0	0	0
Li	2019	141	69.5	40	4	24	96	1	.	.	.	.	.	.	.	.	.	0	1	0	0	0	0	1	0	0	0	0	0	0	0	0	0	0	0
Liao	2019	42	75.5	60	3	12	36	0	.	.	.	.	.	.	.	.	.	1	1	0	0	0	1	0	0	0	0	0	0	0	0	0	0	0	0
Phirom	2020	40	70.2	60	3	12	36	1	.	.	.	.	.	.	.	.	.	1	1	0	0	0	0	0	0	0	0	0	0	0	0	0	0	0	0
Thapa	2020	68	72.6	100	3	8	24	0	1	.	.	.	.	.	.	.	.	0	1	0	0	0	0	0	0	0	0	0	0	0	0	0	0	0	1
Amjad	2019	38	62.8	30	5	6	30	0	.	.	.	.	.	.	.	.	.	1	0	0	0	0	0	1	0	0	0	0	0	0	0	0	0	0	0
Anderson-Hanley	2018	14	75.4	45	2	24	48	.	1	.	.	.	.	.	.	.	.	0	0	0	0	0	0	1	0	0	0	0	0	0	0	0	0	0	0
Jiranan	2020	70	.	60	2	12	24	.	.	.	.	.	.	.	.	.	.	1	1	0	0	0	0	0	0	0	0	0	0	0	0	0	0	0	0
Joke	2017	17	86.9	30	2	6	12	.	.	.	.	.	.	.	.	.	.	0	0	0	0	0	0	1	0	0	0	0	0	1	0	0	0	0	0
Köbe	2016	22	70	45	2	24	48	0	0	0	0	.	0	.	.	.	1	1	1	0	0	0	0	0	0	0	0	0	0	0	0	0	0	0	0
Law	2019	59	71.57	.	.	8	12	1	.	.	1	.	.	.	.	.	.	1	1	0	0	0	0	0	0	0	0	0	0	1	0	0	0	0	0
Park	2020	35	75.8	30	5	6	30	0	.	.	.	.	.	.	.	.	.	0	0	0	0	0	0	0	1	0	0	0	0	0	0	0	0	0	0
Schwenk	2016	20	77.8	45	2	4	8	.	.	.	.	.	.	.	.	.	.	0	0	0	0	0	0	1	0	0	0	0	0	1	0	0	0	0	0
Schimada	2017	266	71.6	90	1	40	40	.	.	.	.	.	.	.	.	.	.	1	1	0	0	0	0	0	0	0	0	0	0	1	0	0	0	0	0
Zijun	2020	11	70.6	60	3	12	.	.	.	.	.	.	.	.	.	.	.	0	1	0	0	0	0	0	0	1	0	0	0	0	0	0	0	0	0
Burgener	2008	43	77	60	3	40	120	1	.	.	.	.	.	.	.	.	.	0	1	0	0	0	0	0	0	1	0	0	0	0	0	0	0	0	0
Cott	2002	86	82	30	5	16	80	.	.	.	.	0	.	.	.	.	.	0	0	0	1	0	0	0	0	0	0	0	0	0	0	0	1	0	0
Friedman and Tappen	1991	30	72.8	30	3	40	120	.	.	.	.	1	.	.	.	.	.	0	0	0	1	0	0	0	0	0	0	0	0	0	0	0	1	0	0
